# Assessment of free radical scavenging and anti-proliferative activities of *Tinospora cordifolia* Miers (Willd)

**DOI:** 10.1186/s12906-017-1953-3

**Published:** 2017-09-11

**Authors:** Picheswara Rao Polu, Udupa Nayanbhirama, Saleemulla Khan, Rajlexmi Maheswari

**Affiliations:** 1 Department of Pharmacognosy, Manipal College of Pharmaceutical Sciences, Manipal University, Manipal, Karnataka -576104 India; 2 Directorate of Research (Health Sciences), Manipal University, Manipal, Karnataka 576104 India

**Keywords:** *Tinospora cordifolia*, Anti-oxidant, Berberine, Cervical carcinoma, SRB assay

## Abstract

**Background:**

*Tinospora cordifolia* (Guduchi or Amrita) is an important drug of Ayurvedic System of Medicine and found mention in various classical texts for the treatment of diseases such as jaundice, fever, diabetes, cancer and skin disease etc. In view of its traditional claims, antioxidant and anti-proliferative activities were evaluated in the present study.

**Methods:**

Ethanol extract (TCE) and subsequent petroleum ether (TCP), dichloromethane (TCD), *n*-Butanol (TCB) and aqueous (TCA) fractions of were prepared from stems of *T cordifolia*. Total phenolic, flavonoid content and anti-oxidant activity was assessed by different methods. Anti-proliferative activity was assessed in cervical carcinoma (HeLa) cell lines by MTT and SRB assay.

**Results:**

Ethanol extract and *n*-butanol fractions shown to be superior in their scavenging activity in all the tested methods. *n*-butanol fractions shown antioxidant activity with an IC_50_ of 14.81 ± 0.53, 29.48 ± 2.23, 58.20 ± 0.70 and 21.17 ± 1.19 μg/mL by DPPH, ABTS, Nitric oxide and iron chelating activities respectively. Anti-proliferative activity results demonstrates that the TCD and ethanol extract of *T cordifolia* exhibits potent cytotoxic effect against HeLa with an IC_50_ of 54.23 ± 0.94 μg/mL and 101.26 ± 1.42 μg/mL respectively by MTT assay; and with an IC_50_ of 48.91 ± 0.33 μg/mL and 87.93 ± 0.85 μg/mL respectively by SRB assay.

**Conclusion:**

The outcomes of the present study support the fact that *T Cordifolia* is a promising source of antioxidant agent and propose its further investigation. Moreover, dichloromethane fraction of *T cordifolia* shown to be the most potent anti-proliferative fraction and further mechanistic and phytochemical investigations are under way to identify the active principles.

## Background

Medicinal plants have been used as a source of medicine with their own personal ways, which have been passed from one generation to another. Owing to their versatile applications plant-derived substances have recently become of great interest [1]. In recent years, because of the concerns on the safety against synthetic drugs the use of natural antioxidants has been promoted [2]. Antioxidant play a major role in normal physiological functions in humans by protecting against cell damage by reactive oxygen species and reducing the adverse effects of these free radicals. Oxidative stress is the major causative factor for producing free radicals and reactive oxygen species (ROS). These free radicles acts as a primary catalysts for initiating oxidation in in vivo and in vitro*,* for creating oxidative stress which leads to numerous diseases and disorder such as cancer, cardiovascular disease, alcohol induced liver disease, neural disorder, alzheimer’s disease, Parkinson’s disease, ageing and atherosclerosis [3].

Phenolic compounds acts as an antioxidant agents, by scavenging the free radicles due to the presence of hydroxyl group in them [4, 5] and they also act as reducing agents, hydrogen donors, metal chelators and singlet oxygen quenchers due to their redox properties [6]. Phenolic acids and flavonoids are considered as typical phenolic compounds that possess antioxidant activity and they were widely distributed in the plant kingdom [7]. The use of synthetic antioxidants have been restricted due to their health risks and toxicity [8]. Rosemary and sage are well known natural antioxidants, exploited commercially either as antioxidant additives or as nutritional supplements demanding the antioxidant potential of plant species [9]. In recent years, the interest in natural antioxidant, especially of plant origin, has greatly increased [10].

Cancer is the second leading cause of death in the world after cardiovascular diseases. Deaths arising from cancer constitute 2–3% of the annual deaths recorded worldwide and kill about 3500 million people annually all over the world. Cervical cancer is the most common cause of cancer death among women in developing countries and in worldwide it is the second most common cancer in women. It is initiated by a change in the epithelial cells, which line the wall of the cervix, and human Papillomavirus (HPV) is the most common risk factor for this type of cancer [11]. A trend to combine conventional therapy with some form of complementary therapy is growing rapidly. Throughout medical history, plant products have been shown to be valuable sources of novel anticancer drugs. There is a widespread use of herbal medicines depicted even in conventional medical history. Out of 121 prescription drugs in use for cancer treatment, 90 are derived from plant species and 74% of these drugs were discovered by investigating a folklore claim [12].

*Tinospora cordifolia* (Willd.) Miers (Family: Menispermaceae) commonly known, as “Amrita” or “Guduchi” is an important drug of Indian Systems of Medicine (ISM) and used in medicines since times immemorial. The drug is well known Indian bitter and prescribed in fevers, diabetes, dyspepsia, jaundice, urinary problems, skin diseases and chronic diarrhoea and dysentery. It has been also indicated useful in the treatment of heart diseases, leprosy, helmenthiasis and rheumatoid arthritis [13]. In the previous study reported, antifungal activity and HPLC analysis were evaluated for the methanolic crude extracts of *T cordifolia* [14]. Antioxidant activity of *T cordifolia* root extract was evaluated in alloxan diabetic rats [15] and same authors also evaluated for hypoglycaemic and other related actions [16]. Phytochemical screening and antimicrobial activities of extracts of *T cordifolia* was evaluated against some pathogenic microbes [17]. Recent study shown that combination of *T cordifolia* and turmeric extracts were effective against the isoniazid, rifampicin, pyrazinamide and ethambutol induced hepatotoxicity, which were used for treating tuberculosis [18]. *T cordifolia* also shown the antitumor and immunomodulatory activities [19, 20]. A novel polysaccharide with immunomodulatory activity was isolated from *T cordifolia* [21]. The plant has been shown to be neuro- protective activity by modulating the antioxidant system in rat hippocampal slices subjected to oxygen glucose deprivation [22].

Hence we aimed to investigate the antioxidant and anti-proliferative effect of stem extract and it’s fractions of *Tinospora cordifolia* against human cervical cancer cells (HeLa) and this is the first preliminary study which employed the stems of this plant as potential source of antioxidant and cytotoxic agents. An attempt was also made to correlate its use as an adjuvant to mitigate oxidative stress in cancer progression.

## Methods

### Plant collection

The stems of *Tinospora cordifolia* were collected from local market of Udupi, Karnataka and the identification of the plant was done by Dr. Gopala Krishna Bhat, Taxonomist, Udupi and the voucher specimen (PP 618) was deposited in the department; the microscopic characteristics of this plant were studied and matched with available literature. The fresh plant material collected was air-dried and grinded to fine powder and stored in air-tight containers for further studies.

### Reagents and chemicals

Folin-Ciocalteu reagent (F9252), gallic acid (G7384), quercetin (Q4951), ascorbic acid (A0278), curcumin (C7727), 1,1-diphenyl-2-picrylhydrazyl radical (DPPH- D9132), 2,2′-azino-bis(3-ethylbenzothiazoline- 6-sulfonic acid) diammonium salt (ABTS- A1888), MTT (3-(4, 5-dimethylthiazol-2-yl)-2, 5-diphenyltetrazolium bromide- M5655) and SRB (Sulforhodamine B- S1402) were purchased from Sigma Aldrich, USA. Methanol, ethanol (95%), dichloromethane, acetonitrile, petroleum ether and DMSO were purchased from Ranbaxy Fine Chemicals Ltd. The reference standard berberine (B3251) was obtained from Sigma Aldrich, USA. All solvents purchased were of analytical grade.

### Preparation of extract and its fractionation

**Fig. 1:**
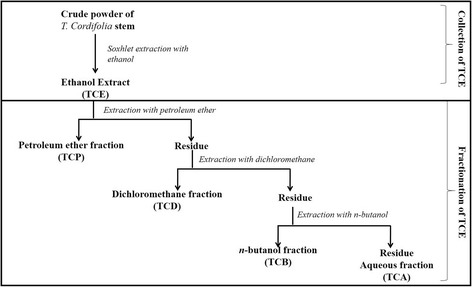
Schematic representation of extraction and fractionation of *T cordifolia*

The shade-dried and coarsely powdered stems of *T cordifolia* (4 Kg) were exhaustively extracted with absolute ethanol by conventional soxhlet extraction at a temperature of 60 °C. After the extraction, under reduced pressure and controlled temperature the extract was concentrated and stored in desiccator for further use. The ethanol (TCE) extract was dissolved in water and partitioned with 3times each of petroleum ether (TCP), Dichloromethane (TCD), *n*-butanol (TCB) and the last fraction was aqueous (TCA) (Fig. 1). All the fractions were evaporated to dryness by using rotary evaporator (IKA RV10- CAT 9.813004) under reduced pressure and controlled temperature and stored for further use. Percentage yield was calculated and reported.

### Phytochemical screening

For the presence of secondary metabolites viz. alkaloids, terpenoids, sterols, phenols, flavonoids, glycosides, tannins, saponins, fixed oils and fats in the extracts/fractions of *T cordifolia* preliminary phytochemical screening was done by using standard tests [23, 24].

### Total phenolic content

The total phenolic content was measured using Folin- Ciocalteu method with slight modification [25]. All samples and readings were prepared and measured in triplicate. Gallic acid was used as standard at concentration ranging from 0.01 mg/mL to 0.05 mg/mL were prepared by diluting the stock solution with distilled water. The extract was prepared at concentration of 1 mg/mL. Briefly, 100 μL of extract was transferred into a test tube and 0.75 mL of Folin-Ciocalteu reagent (previously diluted 10-fold with distilled water) was added and mixed. The mixture was allowed to stand at room temperature for 5 min. Then, 0.75 mL of 6% (*w*/*v*) sodium carbonate was added to the mixture and mixed gently. After standing at room temperature for 90 min, the absorbance was read at 725 nm using UV/Vis spectrophotometer. The standard calibration curve of gallic acid (0.01 mg/mL - 0.05 mg/mL) was plotted to get the R^2^ value and linear equation of gallic acid.

### Total flavonoid content

The total flavonoid content was estimated by aluminium chloride colorimetric method for extracts and fractions of *T cordifolia* [26]. To the reaction test tubes 0.5 ml of stock solution (1 g/ml) of the extract, 1.5 ml methanol, 0.1 ml potassium acetate (1 M) was added and volume was made up to 5 ml with distilled water. After 30 min of incubation at room temperature, the absorbance of the reaction mixture was measured at 415 nm. Total flavonoid content was calculated by extrapolating the absorbance of reaction mixture on standard curve of quercetine. The experiment was repeated thrice and the total flavonoid content was expressed as equivalent to quercetine in mg/g of the extracts.

### Antioxidant activity

#### DPPH radical scavenging activity

The free radical scavenging activity of the extract and fractions of *T cordifolia* was evaluated using the stable free radical i.e., DPPH. 1.0 mL of standard/extract solution at different concentrations was added to 1.0 mL of 0.1 mM DPPH solution in methanol and the absorbance of mixture recorded at 517 nm after 20 min of incubation [27]. Ascorbic acid was used as positive control. DPPH radical scavenging activity was calculated using the formula: Percent scavenging = ((Ao – At)/Ao) × 100; where Ao = Absorbance of control (without extract) and At = Absorbance of sample. All samples and readings were prepared and measured in triplicate.

#### ABTS radical scavenging activity

By reacting, 7 mM ABTS solution with 2.45 mM potassium persulphate, ABTS free radical was generated and it was allowed to stand for 15 h in dark at room temperature. To obtain the absorbance of 0.7 ± 0.2 units at 750 nm, the overnight stored ABTS solution was diluted with methanol. The standard/extract solutions were prepared at various concentrations in methanol and 20 μL of test solutions were added to 180 μL of ABTS free radical solution. The absorbance was measured at 750 nm after 20 min incubation [28]. Ascorbic acid was used as positive control. The ABTS free radical scavenging activity was calculated using the formula: Percent scavenging = ((Ao – At)/Ao) × 100; where Ao = Absorbance of control (without extract) and At = Absorbance of sample. All samples and readings were prepared and measured in triplicate.

#### Nitric oxide scavenging activity

Nitric oxide scavenging activity was estimated by using the Griess reagent assay. Briefly, 0.5 mL of standard/extract solutions at different concentrations were mixed with 2 mL of 10 mM sodium nitroprusside and 0.5 mL phosphate buffered saline (PBS) and. Then the mixture was incubated for 150 min at 25 °C. After the incubation, 0.5 mL of reaction mixture was incubated with 1 mL sulphanilic acid reagent (0.33% sulphanilic acid in 20% glacial acetic acid) for 5 min followed by addition of 1 mL 0.1% naphthyl ethylene diamine dihydrochloride. This incubation mixture was allowed to stand for 30 min and absorbance read at 540 nm. Curcumin was used as positive control [29]. Percentage scavenging was calculated by the following formula: Percent scavenging = ((Ao – At)/Ao) × 100; where Ao = Absorbance of control (without extract) and At = Absorbance of sample. All samples and readings were prepared and measured in triplicate.

#### Iron chelating activity

1, l0-Phenanthroline-iron (III) reagent was prepared by mixing 2 mL of 1 M hydrochloric acid, 0.16 g of ferric ammonium sulphate and 0.198 g of l, l0-phenanthroline monohydrate in 100 mL water. Briefly, 0.2 mL 1, l0- phenanthroline-iron (III) reagent, 0.6 mL methanol and 4 mL water were mixed with 0.2 mL standard/extracts and absorbance measured at 510 nm after the incubation period of 30 min at 50 °C. Ascorbic acid was used as positive control. Higher iron chelating activity was indicated by higher absorbance [30, 31]. Percentage scavenging was calculated by using the following formula: Percent scavenging = = ((Ao – At)/Ao) × 100; where Ao = Absorbance of control (without extract) and At = Absorbance of sample. All samples and readings were prepared and measured in triplicate.

### Total antioxidant capacity by phosphomolybdenum method

The total antioxidant capacities of the extract and fractions of *T cordifolia* were determined using phosphomolybdenum method. Reagent solution was prepared by mixing 0.6 M sulfuric acid, 28 mM sodium phosphate and 4 mM ammonium molybdate. 1 mL of reagent solution was added to 0.1 mL of sample solutions (1 mg/mL) and then those tubes were capped and incubated for 90 min at 95 °C in a boiling water bath. The absorbance of the solution of each was measured at 695 nm against blank after the samples had cooled down to room temperature. Antioxidant capacity of samples were expressed as equivalents of ascorbic acid (mg/g of extract) [32].

### Cell culture

Human cervical cancer cells, HeLa were obtained from National Centre for Cell Science, Pune, India. By using Dulbecco’s Minimum Essential Medium (DMEM- D5796) with 10% fetal bovine serum (FBS- F2442) and 50 μg/mL gentamicin cells were cultured. The cells were grown by incubating at 37 °C with humidified atmosphere by providing 5% CO_2_ and 95% air in CO_2_ incubator (CAT 51030387- Thermo Fisher Scientific). By using inversion microscope to see the morphology and cell growth, while ensuring that no contamination occurs in a culture flask the cultured cells were observed and checked daily. The cells were sub-cultured up to 70% - 90% collisions between the cells. For cell viability assay, cells which are in their exponential phase were used.

### Cell viability by MTT assay

**MTT assay:** from 75 cm^2^ tissue culture flasks HeLa cells were collected and a stock cell suspension (1 × 10^5^ cells/ml) was prepared. A 96-well plate was seeded with 0.1 ml of DMEM medium and supplemented by adding 10% FBS and allowed to attach for 24 h. Just prior to the experiment, test compounds were dissolved in 0.1% DMSO. Cells were treated with 20 μL of test solutions from respective stocks (25, 50, 100 and 200 μg/mL) after 24 h of incubation and a fresh medium of 80 μL was added and incubated for 48 h. The medium containing 0.1% DMSO alone served as the control. Doxorubicin was used as standard. After the treatment drug containing media was removed and washed with 200 μL of PBS. After adding100μL of MTT reagent, cells were incubated for 4 h at 37 °C. After incubation, MTT reagent was removed by inverting the plate and formazan crystals were solubilized by adding 100 μL of 100% DMSO [33]. An ELISA plate reader at 540 nm was used to measure the optical density (O.D); followed by the calculation of percentage viability. Percentage cell viability = 100 - [((Ao-At)/Ao) × 100], where Ao = Absorbance of cells treated with 0.1% DMSO medium, At = Absorbance of cells treated with extract/fractions. Experiment was done in triplicates; Results were expressed as Mean ± SEM values (proportional to cell survival) and graphs were plotted against the drug concentrations which were tested for cytotoxicity.

**SRB assay:** 100 μL of cell suspension of HeLa was introduced into each well of 96-well tissue culture plate. Cells were treated with100 μL of various concentrations (25, 50, 100 and 200 μg/mL) of the test solution and incubated for 48 h. The medium containing 0.1% DMSO only served as control and Doxorubicin was used as standard. After incubation, cells were fixed by treating with ice cold TCA for 1 h at 40 °C. Plates were washed and allowed for drying. Cells were subjected for staining at room temperature for 30 min by adding 50 μL of SRB solution. 1% *v*/v acetic acid was added to remove unbound SRB and allowed to dry. 100 μL of 10 mM unbuffered Tris Base (pH 10.5) was added to solubilize the bound SRB and the plate was kept on a shaker platform for 5 min [34]. An ELISA plate reader at 570 nm was used to measure the optical density (O.D); followed by the calculation of percentage viability. Percentage cell viability = 100 - [((Ao-At)/Ao) × 100], where Ao = Absorbance of cells treated with 0.1% DMSO medium, At = Absorbance of cells treated with extract/fractions. The IC_50_ values were determined by plotting O.D values against the tested concentrations of the drug. Experiment was done in triplicates; Results were expressed as Mean ± SEM values (proportional to cell survival) were plotted against the drug concentrations which were tested.

### Quantitative analysis of berberine in extract/ fractions by HPTLC

To detect and quantify the content of berberine in extract/fractions of *T cordifolia* a validated high performance thin layer chromatography (HPTLC) method was used [35]. Extract/fractions prepared at concentration of 2 mg/mL in methanol and standard solutions were prepared at a concentration of 100 μg/mL. The plates were pre-washed with methanol before spotting. By using Camag Linomat V sample applicator sharp bands of standard and sample solutions were applied on the plates and dried by current of hot air. The mobile phase 20 mL (Chloroform: methanol: water (8:2:0.2, *v*/v) was poured in a twin trough glass chamber and whole assembly was left for 30 min for the saturation**.** Applied plate was placed in the chamber after 30 min and then developed until the solvent front had travelled at a distance of 80 mm above the base of the plate. Then the plate was removed from the chamber and dried in a current of hot air. By using Camag TLC Scanner 3 at a wavelength of 350 nm detection of bands and its quantification was performed.

### Statistical analysis

The results are expressed as mean ± standard error of mean (SEM) of three replicate determinations and then analyzed by Graph pad prism 5. One way analysis of variance (ANOVA) and post-hoc Tukeys test were used to determine the differences among the means. A value of *P* < 0.05 was considered to be statistically significant.

## Results & discussion

In this study we evaluated the antioxidant and anti-proliferative activities of the total extract and subsequent fractions of *T cordifolia* in human cervical cancer cancer cells. In addition, the antioxidant activity and phytochemical analysis was carried out for TCE/fractions to further its use as an antioxidant and chemopreventive agent.

### Phytochemical analysis

The extraction yield, total phenolic and flavonoid contents in the extract and fractions of *T cordifolia* were shown in Table 1.

**Table 1:** Percent yield, total phenolic and flavonoid content of extract and fractions of *T cordifolia*

S.No	Extract/Fraction	Percentage yield (%w/w)	Total phenolic content (mg GAE/g of plant extract)	Total Flavonoid content (mg QE/g of plant extract)
1	TCE	7.4	4.2 ± 0.25^a^	0.52 ± 0.02 ^a^
2	TCP	19.08	1.1 ± 0.04 ^b^	0.98 ± 0.01 ^b^
3	TCD	17.90	2.4 ± 0.39 ^c^	0.19 ± 0.10 ^c^
4	TCB	16.21	5.1 ± 0.18 ^d^	0.25 ± 0.02 ^d^
5	TCA	42.56	1.8 ± 0.16 ^e^	0.17 ± 0.08 ^e^

All the values are expressed as mean ± SEM (*n* = 3)

^a-e^Column wise values with different superscripts of this type indicate significant difference (*P* < 0.05)

The presence of sterols, triterpenoids, phenols, flavonoids, alkaloids, saponins, tannins, fixed oils and fats in ethanol extract (TCE) of *T cordifolia* was confirmed by preliminary phytochemical screening. The presence of sterols and triterpenoids in TCP and TCD was confirmed by Liebermann Burchard and Salkowski’s tests and the presence of phenols in TCB and TCA was confirmed by the ferric chloride test. The presence of flavonols (isoflavones), flavones, flavonones, flavononols in TCD and TCB was confirmed by positive results from Shinoda test and the presence of alkaloids in TCB and TCA was confirmed by Dragendorff and Mayer’s tests.

Phenolic compounds acts as an antioxidant agents, by scavenging the free radicles due to the presence of hydroxyl group in them and they can act as reducing agents, hydrogen donors, metal chelators and singlet oxygen quenchers due to their redox properties [36]. TCB (5.1 ± 0.18 mg GAE/g of plant extract) contains the maximum amount of phenolics which was found to be significantly higher (*P* < 0.05) than other plant fractions and the non-polar TCP contain the least amount of phenolics which was found to be 1.1 ± 0.04 mg GAE/g of plant extract. Results were calculated from standard gallic acid calibration curve with R^2^ value of 0.9960.

Flavonoids form the largest group of natural phenolic compounds and possess excellent free radical scavenging and antioxidant properties [37]. By using the aluminium chloride method total flavonoid content was determined using a linear calibration curve of quercetin with R^2^ value of 0.9980. TCP was found to be contain the maximum amount of flavonoids (0.98 ± 0.01 mg QE/g of plant extract) and TCA contain the least amount of flavonoids (0.17 ± 0.08 mg QE/g of plant extract).

### Antioxidant activity

**Fig. 2:**
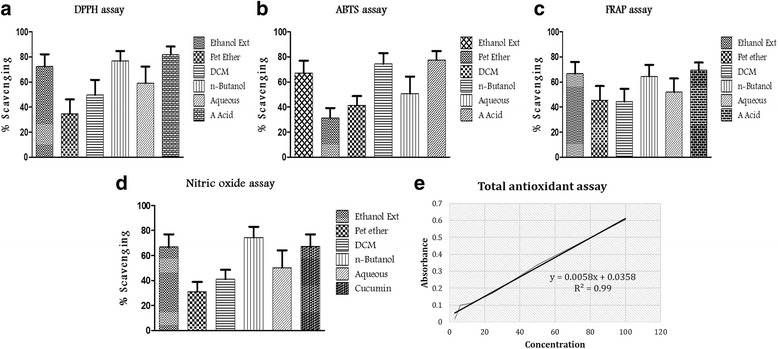
In vitro antioxidant assays of *T cordifolia.* Antioxidant activities of ethanol extracts and its fractions of *T cordifolia.* IC_50_ values were calculated by mean percentage of Scavenging at different concentrations (12.5, 25, 50,100 and 200 μg/mL). **a**) DPPH assay **b**) ABTS assay **c**) FRAP assay **d**) Nitric oxide assay **e**) Total antioxidant capacity

Antioxidants may impart a defensive role by three main proposed mechanisms: hydrogen atom transfer, single electron transfer and metal chelation [38]. Reactive Oxygen/Nitogen Species have a main role in the endogenous defensive system, an imbalance in these system leads to oxidative stress and damage to cellular molecules leading to carcinogenesis [39]. Due to the presence of phenols and flavonoids in TCE/fractions, we evaluated the free radical scavenging activity using suitable in vitro models, which would further its use as an adjuvant in chemoprevention and the observed different antioxidant activities of the extracts opposed to different assay models may be attributed to the different mechanisms of the radical-antioxidant reactions (Fig. 2).

### DPPH free radical scavenging activity

DPPH is often used to determine free radical scavenging activity of natural compounds due to its stability as a radical [40]. The presence of unpaired electron imparts a strong absorbance at 517 nm, giving the radical a purple color. With the exposure to antioxidants, it undergoes reduction, decreasing absorbance due to the formation of yellow coloured anti-radical diphenylpicryl hydrazine (DPPH-H). The degree of colour change from purple to yellow is a measure of scavenging potential of the antioxidants in the extracts in terms of hydrogen donating ability [41]. In the current study, all extract/fractions and positive control (ascorbic acid) exhibited a significant, dose-dependent radical scavenging activity (*p* < 0.05). The IC_50_ values of extracts were found within the range of 2.57 ± 0.31–183.47 ± 2.20 μg/mL. The *n-*butanol fraction of *T cordifolia* shown a marked DPPH free radical scavenging activity with an IC_50_ values of 14.18 ± 0.53 μg/mL, and the ethanol extracts and other fractions of *T cordifolia* were also found to be more effective as DPPH free radical scavengers in comparison with the reference standard, ascorbic acid. Petroleum ether fraction showed the least free radical scavenging activity (Table 2).

**Table 2:** Free radical scavenging and antioxidant capacity of extract and fractions of *T cordifolia*

Extract/Fraction	DPPH scavenging IC_50_ (μg/mL)	ABTS scavenging IC_50_ (μg/mL)	Nitric oxide scavenging IC_50_ (μg/mL)	Iron chelation IC_50_ (μg/mL)	Total antioxidant capacity^f^
TCE	16.87 ± 0.08^a^	28.24 ± 1.83^a^	86.09 ± 0.74^a^	29.70 ± 1.08^a^	34.46 ± 1.37^a^
TCP	183.47 ± 2.20^b^	>1000	491.57 ± 1.25^b^	54.94 ± 2.97^b^	53.27 ± 2.24^b^
TCD	95.96 ± 0.62^c^	132.4 ± 1.14^b^	306.25 ± 1.29^c^	22.25 ± 0.46^a^	34.33 ± 2.27^a^
TCB	14.18 ± 0.53^a^	29.48 ± 2.233^a^	58.20 ± 0.70^a^	21.17 ± 1.19^c^	33.57 ± 1.17^a^
TCA	74.25 ± 2.14^d^	92.46 ± 2.06^c^	182.14 ± 1.03^d^	46.23 ± 0.34^d^	39.92 ± 0.9^a^
Ascorbic acid	2.57 ± 0.31^e^	4.17 ± 0.29^d^	–	1.82 ± 0.07^e^	–
Curcumin	–	–	15.03 ± 0.79^e^	–	–

All the values are expressed as mean ± SEM (*n* = 3)

^a-e^Column wise values with different superscripts of this type indicate significant difference (*P* < 0.05). f-Total antioxidant capacity expressed as μg ascorbic acid equivalents/mg extract

### ABTS radical scavenging activity

In this assay, the capability of antiradical elements to satisfy the ABTS_+, a blue–green chromophore with attribute absorption at 734 nm. The inclusion of antioxidants to the conducted radical cation decreases it to ABTS, identifying a decolourization. In this technique, an antioxidant is included in a pre-formed ABTS radical solution, and after a set period, the staying ABTS _ + is quantified spectrophotometrically at 734 nm [42, 43]. In the current study, all extract/fractions except petroleum ether fraction and positive control (ascorbic acid) exhibited a significant, dose-dependent radical scavenging activity (*p* < 0.05). The IC_50_ values of extracts were found within the range of 4.17 ± 0.29–132.14 ± 1.14 μg/mL. *n-*butanol fraction shown a marked ABTS free radical scavenging activity with an IC_50_ values of 29.48 ± 2.23 μg/mL, and the ethanol extract and other fractions of *T cordifolia* were also found to be more effective as ABTS free radical scavengers in comparison with the reference standard, ascorbic acid. Petroleum ether fraction not shown free radical scavenging activity (Table 2).

### Nitric oxide scavenging activity

In several inflammatory diseases and carcinogenesis Nitric oxide plays an important role and it is generated from the amino acid L-arginine by vascular endothelial cells, phagocytes and certain cells of brain. It is classified as free radical, because of its unpaired electron and displays important reactivity with certain types of proteins and other free radicals. The toxicity of NO becomes adverse when it reacts with superoxide radical, forming a highly reactive peroxynitrite anion (ONOO^−^) [44]. In this assay, nitric oxide generated from sodium nitroprusside reacts with oxygen to form nitrite. The nitrite ion diazotize with sulphanilamide acid and couple with naphthyl ethylenediamie, forming pink colour which was measured at 546 nm [45]. As antioxidants donates protons to the nitrite radicle, the absorbance was used to measure the extent of nitrite radicle scavenging [46]. In the current study, all extract/fractions and positive control (ascorbic acid) exhibited a significant, dose-dependent radical scavenging activity (*p* < 0.05). The IC_50_ values of extracts were found within the range of 15.03 ± 0.79–491.57 ± 1.25 μg/mL. *n-*butanol fraction shown a marked nitric oxide free radical scavenging activity with an IC_50_ values of 58.20 ± 0.70 μg/mL, and the ethanol extracts and other fractions of *T cordifolia* were also found to be more effective as nitric oxide free radical scavengers in comparison with the reference standard, ascorbic acid. Petroleum ether fraction showed the least free radical scavenging activity (Table 2).

### Iron chelation activity

Iron ions are known to catalyze the conversion of less reactive species like H_2_O_2_ or lipid peroxides to more reactive ones such as hydroxyl, peroxyl/alkoxyl radicals. The release of iron by cellular damage can accelerate oxidative damage hence, compounds with iron chelating ability can act as powerful antioxidants [47]. In the current study, all extract/fractions and positive control (ascorbic acid) exhibited a significant, dose-dependent radical scavenging activity (*p* < 0.05). The IC_50_ values of extracts were found within the range of 1.82 ± 0.07–54.94 ± 2.97 μg/mL. The *n-*butanol fraction shown a marked iron chelating activity with an IC_50_ values of 21.17 ± 1.19 μg/mL, and the ethanol extract and other fractions of *T cordifolia* were also found to be more effective as iron chelators in comparison with the reference standard, ascorbic acid. Petroleum ether fraction showed the least free radical scavenging activity (Table 2).

### Total antioxidant capacity

Total antioxidant activity of extract and fractions were analyzed by the formation of phosphomolybdenum (PM) complex based on the reduction degree of Mo (VI) to Mo (V). It is a quantitative method to investigate the reduction reaction among antioxidant, oxidant and molybdenum ligand by involving in thermally generating auto-oxidation during prolonged incubation at higher temperature. TCB shown maximum antioxidant capacity equal to 33.57 ± 1.17 AAE/mg extract followed by TCD, TCE, TCA and TCP (Table 2).

### Anti-cancer activity

Phyto-compounds have been used as an outstanding source of drug leads from several decades due to their unique nature and their wide range of structural diversity and are “biologically friendly” by Mother Nature [48]. A study reported recently by the European anticancer drug market states that 155 anti-tumor drugs accepted clinically of which 47% were obtained from natural source or derivative products from them [49]. Recently, Ahmad S et al., reported the cytotoxic potential of *Rumax hastatus* against HeLa cell lines [50].

### Anti-proliferative activity of TCE/fractions in HeLa cells by MTT assay

**Fig. 3:**
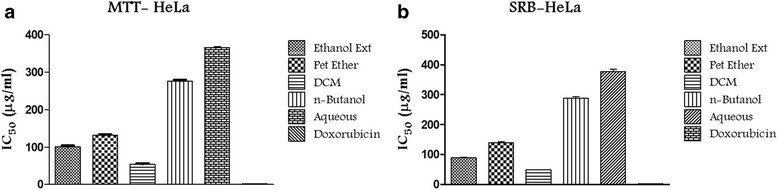
In vitro MTT and SRB assays on HeLa cells. HeLa cells were incubated with different concentrations (25, 50, 100, 200 μg/mL) of ethanol extract and its fractions of *T cordifolia*; Growth inhibition was determined by the MTT and SRB assays. IC_50_ values were calculated by mean percentage of cell death at different concentrations. **a**) MTT assay and **b**) SRB assay

MTT assay is an established method of determining viable cell number in proliferation and cytotoxicity studies [51]. In the present study, cytotoxic effect of the sub fractions of TCE on human cervical cancer (HeLa) cells were determined based on reduction of the yellow colored water soluble tetrazolium dye 3-[4, 5-dimethylthiazol-2-yl]-2,5-diphenyl tetrazolium bromide (MTT) to formazan crystals. Mitochondrial dehydrogenase produced by live cells reduces MTT to blue formazan product, which reflects the normal function of mitochondria and cell viability [52]. The cytotoxic potential results visibly demonstrates that the dichloromethane fraction and ethanol extract of *T cordifolia* exerts strong cytotoxicity against HeLa with an IC_50_ of 54.23 μg/mL and 101.26 μg/mL. The extract and fractions shown dose-dependent inhibition of cell proliferation in HeLa cell line. On the other hand successive fractions of ethanol extract which includes, petroleum ether, *n*-butanol and water fractions, shown relatively distinct cytotoxic effect (Tables 3 and 4 Fig. 3). Similar cytotoxic activity between extract and its fractions was obtained by Tukey’s post hoc tests study; and doxorubicin as a standard was indicated in Tables 3 and 4.

**Table 3:** Cytotoxicity results of *T cordifolia* on HeLa cell line. Cytotoxicity results of *T cordifolia* by MTT assay on HeLa (Human cervical carcinoma)

S.No	Concentration (μg/mL)	% Cell Death					
		TCE	TCP	TCD	TCB	TCA	Doxorubicin
1	0.01	–	–	–	–	–	39.81 ± 1.06^**^
2	0.1	–	–	–	–	–	48.24 ± 0.27^**^
3	1	–	–	–	–	–	68.43 ± 1.02^**^
4	10	–	–	–	–	–	82.54 ± 0.92^**^
5	25	39.92 ± 0.36^*^	29.24 ± 0.64	55.02 ± 0.68^**^	3.63 ± 1.54	13.47 ± 1.88	–
6	50	56.06 ± 0.73^**^	53.34 ± 0.48^**^	55.95 ± 0.43^**^	29.79 ± 1.79	25.84 ± 1.51	–
7	100	66.76 ± 0.51^**^	66.00 ± 1.12^*^	73.90 ± 0.59^**^	40.95 ± 0.69^*^	28.58 ± 1.98	–
8	200	80.97 ± 1.71^**^	77.30 ± 0.33^*^	81.60 ± 0.28^**^	76.54 ± 0.58^*^	84.02 ± 0.46^**^	–
9	IC_50_	101.26 ± 1.42^b^	131.94 ± 1.23^c^	54.23 ± 0.94^a^	276.03 ± 2.00	366.05 ± 1.74	1.89 ± 0.02^a^

Results were expressed as Mean ± SEM (*n* = 3) and analysed using one-way ANOVA followed by Tukey’s post hoc test of significance [where different alphabets denote significant difference (*p* < 0.05)]. Statistical comparison between the mean % viability of each extract and its negative control was performed using one-way ANOVA followed by Dunnett’s post hoc test of significance wherein *p < 0.05 and **p < 0.01 were considered to be statistically significant and very statistically significant compared to negative control. *T cordifolia* extracts were tested at concentrations of 200, 100, 50, 25 μg/mL and Doxorubicin was tested at concentrations of 0.01, 0.1, 1 and 10 μg/mL

**Table 4:** Cytotoxicity results of *T cordifolia* on HeLa cell line. Cytotoxicity results of *T cordifolia* by SRB assay on HeLa (Human cervical carcinoma)

S.No	Concentration (μg/mL)	% Cell Death					
		TCE	TCP	TCD	TCB	TCA	Doxorubicin
1	0.01	–	–	–	–	–	44.17 ± 0.38^**^
2	0.1	–	–	–	–	–	47.40 ± 0.17^**^
3	1	–	–	–	–	–	69.53 ± 0.02^**^
4	10	–	–	–	–	–	83.45 ± 0.18^**^
5	25	44.58 ± 0.19^*^	27.04 ± 0.95^*^	58.01 ± 0.11^**^	4.42 ± 3.68	10.64 ± 4.20	–
6	50	55.92 ± 0.71^*^	49.62 ± 0.35	59.46 ± 0.04^**^	27.50 ± 0.18	19.70 ± 2.62	–
7	100	69.84 ± 0.24^**^	58.60 ± 0.17^*^	76.68 ± 0.21^**^	42.10 ± 3.02^*^	33.42 ± 4.00	–
8	200	82.50 ± 0.83^**^	75.47 ± 0.12^**^	83.72 ± 0.19^**^	68.21 ± 2.45^*^	80.02 ± 0.38^**^	–
9	IC_50_	87.93 ± 0.85^b^	139.54 ± 1.54^c^	48.91 ± 0.33^a,b^	283.91 ± 2.93	377.39 ± 1.09	1.85 ± 0.02^a^

Results were expressed as Mean ± SEM (*n* = 3) and analysed using one-way ANOVA followed by Tukey’s post hoc test of significance [where different alphabets denote significant difference (*p* < 0.05)]. Statistical comparison between the mean % viability of each extract and its negative control was performed using one-way ANOVA followed by Dunnett’s post hoc test of significance wherein *p < 0.05 and **p < 0.01 were considered to be statistically significant and very statistically significant compared to negative control. *T cordifolia* extracts were tested at concentrations of 200, 100, 50, 25 μg/mL and Doxorubicin was tested at concentrations of 0.01, 0.1, 1 and 10 μg/mL

### Anti-proliferative activity of TCE/fractions in HeLa cells by SRB assay

In order to confirm the findings of MTT assay, SRB assay was carried out in HeLa cells since some phytochemicals are known to interfere with MTT giving false positive results [53]. Based on the measurement of cellular protein content, a unique method called Sulforhodamine B (SRB) assay to determine the cytotoxicity was developed by Skehan and his coworkers [54]. The dichloromethane fraction and ethanol extract of *T cordifolia* exerts strong cytotoxicity against HeLa with an IC_50_ of 48.91 μg/mL and 87.93 μg/mL. Inhibition of tumor cell growth was in dose-dependent manner by both ethanol extract and dichloromethane fractions of *T cordifolia*. On the other hand successive fractions of ethanol extract which includes, petroleum ether, *n*-butanol and water fractions, shown relatively distinct cytotoxic effect (Table 4 and Fig. 3).

The results obtained by MTT and SRB assays illustrate that they were very much similar. To some extent higher IC_50_ values by MTT assay and on the other hand IC_50_ values of ethanol extract and dichloromethane fraction, by SRB assay were found to be somewhat lesser than MTT assay. There was good correlation between the IC_50_ values attained by both MTT and SRB assays, as a whole. Thus we conclude that *T cordifolia* extract and fractions hold effective anti-proliferative activity.

### Quantification of berberine in TCE/fractions by HPTLC

**Fig. 4:**
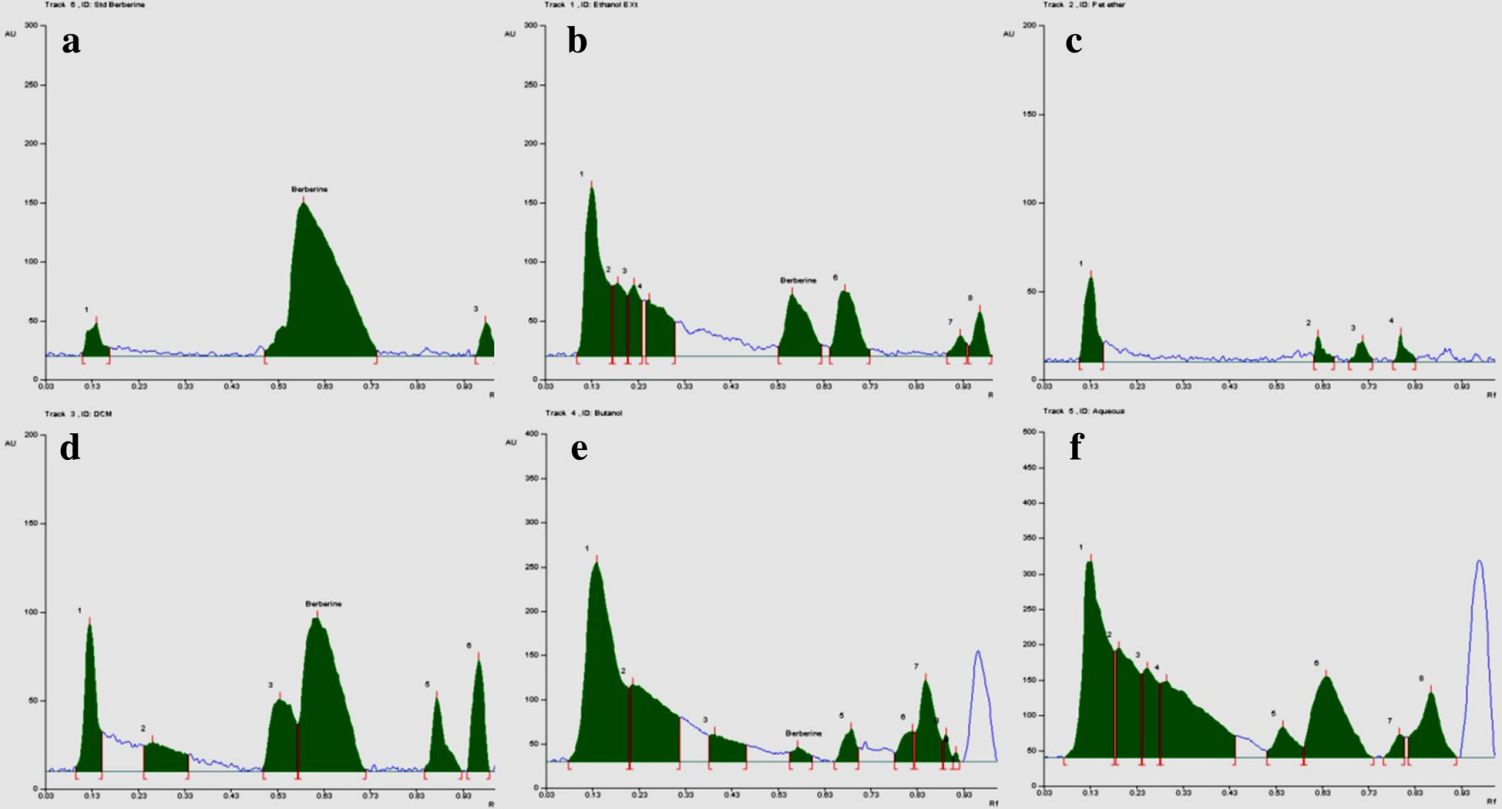
Estimation of berberine in *T cordifolia* ethanol extract and its fractions. Representative chromatograms illustrate High Performance Thin Layer Chromatography (HPTLC) of berberine. (**a**) Standard berberine **b**) ethanol extract (TCE) (**c**) Petroleum ether fraction (TCP) (**d**) Dichloromethane (TCD) fraction **e**) n-Butanol fraction (TCB) and **f**) Aqueous fraction (TCA) . Scanned at 350 nm and berberine eluted at R_f_ value of 0.57

HPTLC analysis of the extract/fractions of *T cordifolia* confirmed the presence of berberine. Total TCE was found to contain 0.42% *w*/w of berberine. Among the fractions, TCD and TCB were found to be enriched with 1.09% and 0.19% w/w and berberine was not detected in TCP and TCA (Table 5 and Fig. 4). Our results were in accordance with previous studies that have reported the presence of berberine in *T cordifolia*.

**Table 5:** Estimation of berberine in *T cordifolia* extract/fractions by HPTLC

S.No	Sample	R_f_ Value	Peak Area	Percentage Content (%w/w)
1	Berberine	0.57	23,546.6	95.24
2	Ethanol Extract	0.57	2112.4	0.42
3	DCM fraction	0.57	5405.1	1.09
4	n-Butanol fraction	0.57	958.1	0.19

Some studies reported that berberine is a natural isoquinoline alkaloid used for eras in different folklore medicines [55], has shown in vitro anti-proliferative and cytotoxic activity against human breast cancer (MCF-7), human cervical cancer (HeLa) and leukemia (L1210) cells, it also shown to be cytotoxic in human colorectal cancer cells (HCT-116), human gastric carcinoma (SNU-5), prostate cancer ((LNCaP) and human adenocarcinoma (HepG2) cell [56], Remarkably, it has shown not the same effects on the cell cycle, with cell cycle arrest G_1_ phase and arrest at G_2_/M phase of the cell cycle along with the no effect on cell cycle, depending on the cell line type used for the study [57]. Kettmann V et al., reported that in cervical cancer (HeLa) and leukemia (L1210) cell lines berberine induces the cell death by DNA topoisomerase I poisoning [58]. Hence, the anticancer activity of the whole extract TCE and TCD fraction against cervical cancer cells could be attributed to the presence of a variety of compounds along with the berberine. Currently, further detailed characterization is being carried out in our laboratory to identify the active compounds and further study their mechanisms of action in cervical cancer.

## Conclusion

The present study has revealed that the ethanol extract of *T cordifolia* contains substantial amount of phenolics and thus, can be inferred that these phenolics are responsible for its marked antioxidant activity as assayed through various in vitro models used in this study. This is consistent with several reports that have shown close relationship between total phenolic contents and antioxidant activity of fruits, plants and vegetables. Therefore, *T cordifolia* stem extracts have considerable antioxidant properties and the consumption of this plant may play a role in preventing human diseases in which free radicals are involved, such as cancer, cardiovascular disease, and premature aging. However, further investigations on the in vivo antioxidant activity and the different antioxidant mechanisms are warranted. Besides, that the ethanol extract and dichloromethane fractions of *T cordifola* shown significant anti-proliferative activity in HeLa (cervical carcinoma)) cell line. So, it can be concluded that *T cordifoila* has the potential to be established as a chemo-preventive option for cancer. However, at this stage it is very difficult to accomplish the possible mechanism. So, the molecular mechanism along with the isolation of phyto-constituents responsible for its anti-cancer activity will be doing in further studies along with the screening for cytotoxicity in other available cancer cell lines.

## Abbreviations

ABTS: 2,2′-azino-bis(3-ethylbenzothiazoline- 6-sulfonic acid) diammonium salt
ANOVA: One way analysis of variance
DMEM: Dulbecco’s minimum essential medium
DMSO: Dimethyl sulfoxide
DPPH: 1,1-diphenyl-2-picrylhydrazyl
FBS: Fetal bovine serum
GAE: Gallic acid equivalents
MTT: 3-(4,5-dimethylthiazolyl-2-yl)-2,5- diphenyl tetrazolium bromide
QE: Quercetin equivalents
RNS: Reactive nitrogen species
ROS: Reactive oxygen species
SRB: Sulforhodamine B
TCA: Aqueous fraction of *T cordifolia*
TCB: *n-*butanol fraction of *T cordifolia*
TCD: Dichloromethane fraction of *T cordifolia*
TCE: Ethanol extract of *T cordifolia*
TCP: Petroleum ether extract of *T cordifolia*
